# Eosinophilic Rhinosinusitis With Nonsteroidal Anti-inflammatory Drug (NSAID)-Exacerbated Respiratory Disease: A Case Report of Sodium Phosphate Corticosteroids-Induced Complications

**DOI:** 10.7759/cureus.79783

**Published:** 2025-02-27

**Authors:** Shigekazu Yoshida, Takuro Okada, Toru Kuwazawa, Shun Mochida, Yasuo Ogawa, Kiyoaki Tsukahara

**Affiliations:** 1 Otolaryngology - Head and Neck Surgery, Tokyo Medical University Hachioji Medical Center, Tokyo, JPN; 2 Otolaryngology - Head and Neck Surgery, Tokyo Medical University, Tokyo, JPN

**Keywords:** eosinophilic chronic rhinosinusitis, hydrocortisone sodium phosphate, nsaid-exacerbated respiratory disease, sodium phosphate corticosteroids, sodium succinate corticosteroids

## Abstract

Nonsteroidal anti-inflammatory drug (NSAID)-exacerbated respiratory disease (N-ERD) is a hypersensitivity disorder characterized by asthma, nasal polyposis, and NSAID intolerance. Eosinophilic chronic rhinosinusitis (ECRS) coexists with N-ERD in approximately 7.4% of cases. As sodium succinate corticosteroids (SSC) may exacerbate respiratory symptoms in patients with N-ERD, sodium phosphate corticosteroids (SPC) are generally recommended as an alternative. This report presents a case in which SPC additives possibly triggered an N-ERD attack during surgery in a patient with ECRS and bronchial asthma. A 58-year-old woman with a history of bronchial asthma was suspected of having ECRS and underwent surgery for diagnostic and therapeutic purposes. During the procedure, a rapid intravenous injection of hydrocortisone sodium phosphate (HSP) led to complete ventilation failure. Postoperative skin prick testing confirmed a positive reaction to HSP. Although anaphylactic shock was initially suspected, the possibility that additives in HSP induced N-ERD attack was also considered. N-ERD is primarily induced by COX-1 inhibitors, but additives such as parabens may also trigger the condition. Furthermore, rapid intravenous administration of corticosteroids has been reported to exacerbate asthma, potentially contributing to this patient’s reaction. Since N-ERD is typically acquired later in life, it should be considered even in patients with no prior history of NSAID-induced attacks, particularly when factors such as severe asthma and olfactory dysfunction are present. This case highlights the importance of selecting suitable medications in patients with N-ERD to prevent life-threatening attacks.

## Introduction

Nonsteroidal anti-inflammatory drug (NSAID)-exacerbated respiratory disease (N-ERD), previously known as aspirin-induced asthma, is a clinical syndrome characterized by hypersensitivity to aspirin and other NSAIDs, nasal polyposis, and asthma [1]. This condition is now referred to as aspirin-exacerbated respiratory disease (AERD) or N-ERD. In this article, we use the term N-ERD since the symptoms are not limited to aspirin.

Chronic rhinosinusitis (CRS) is an inflammatory disorder of the nasal cavity and paranasal sinuses characterized by nasal congestion, nasal discharge, and persistent post-nasal drip for over 12 weeks. Traditionally, CRS was classified based on the presence or absence of nasal polyps, distinguishing CRS with nasal polyps (CRSwNP) from CRS without nasal polyps (CRSsNP) [2]. However, the European Position Paper on Rhinosinusitis and Nasal Polyps (EPOS) 2020 introduced a new classification system, classifying CRS into primary and secondary types and further categorizing each as localized or diffuse based on the affected regions. Primary CRS is classified into type 2 and non-type 2 inflammation. Type 2 inflammation is an allergic response driven by cytokines such as IL-4, IL-5, and IL-13. CRS subtypes associated with type 2 inflammation include eosinophilic CRS (ECRS), CRSwNP, allergic fungal rhinosinusitis (AFRS), and central compartment allergic disease (CCAD) [3].

ECRS, first identified in Japan, is characterized by nasal polyp formation and eosinophilic infiltration of the nasal mucosa [4]. The Japanese Epidemiological Survey of Refractory Eosinophilic Chronic Rhinosinusitis (JESREC), a multicenter study conducted in 2015, established diagnostic criteria for ECRS based on the JESREC score. This scoring evaluates unilateral or bilateral disease, nasal polyps, blood eosinophilia, and ethmoid sinuses involvement on computed tomography (CT) scans, with a cutoff value of 11 points. ECRS is defined histologically by ≥70 eosinophils per high-power field (HPF) in the submucosa of the ethmoid sinus or within nasal polyps [5]. ECRS often coexists with other respiratory conditions, with 26.9% of patients with ECRS in Japan having bronchial asthma and 7.4% having N-ERD [5]. Outside Japan, no reports have specifically focused on ECRS, though CRSwNP is complicated with bronchial asthma in 46.9% of cases [6] and with N-ERD in 16% of cases [7]. The reported prevalence of N-ERD among bronchial asthma patients ranges from 10-20% [8], suggesting a high rate of overlap between bronchial asthma and N-ERD.

When managing N-ERD, sodium phosphate corticosteroids (SPC) are preferred over sodium succinate corticosteroids (SSC), which may exacerbate respiratory symptoms [9]. This report presents a case in which an asthma attack occurred during surgery in a patient with coexisting ECRS and bronchial asthma. An N-ERD reaction was suspected to have been induced by the additives in SPC. This case is presented in the context of relevant literature.

## Case presentation

**Figure 1:**
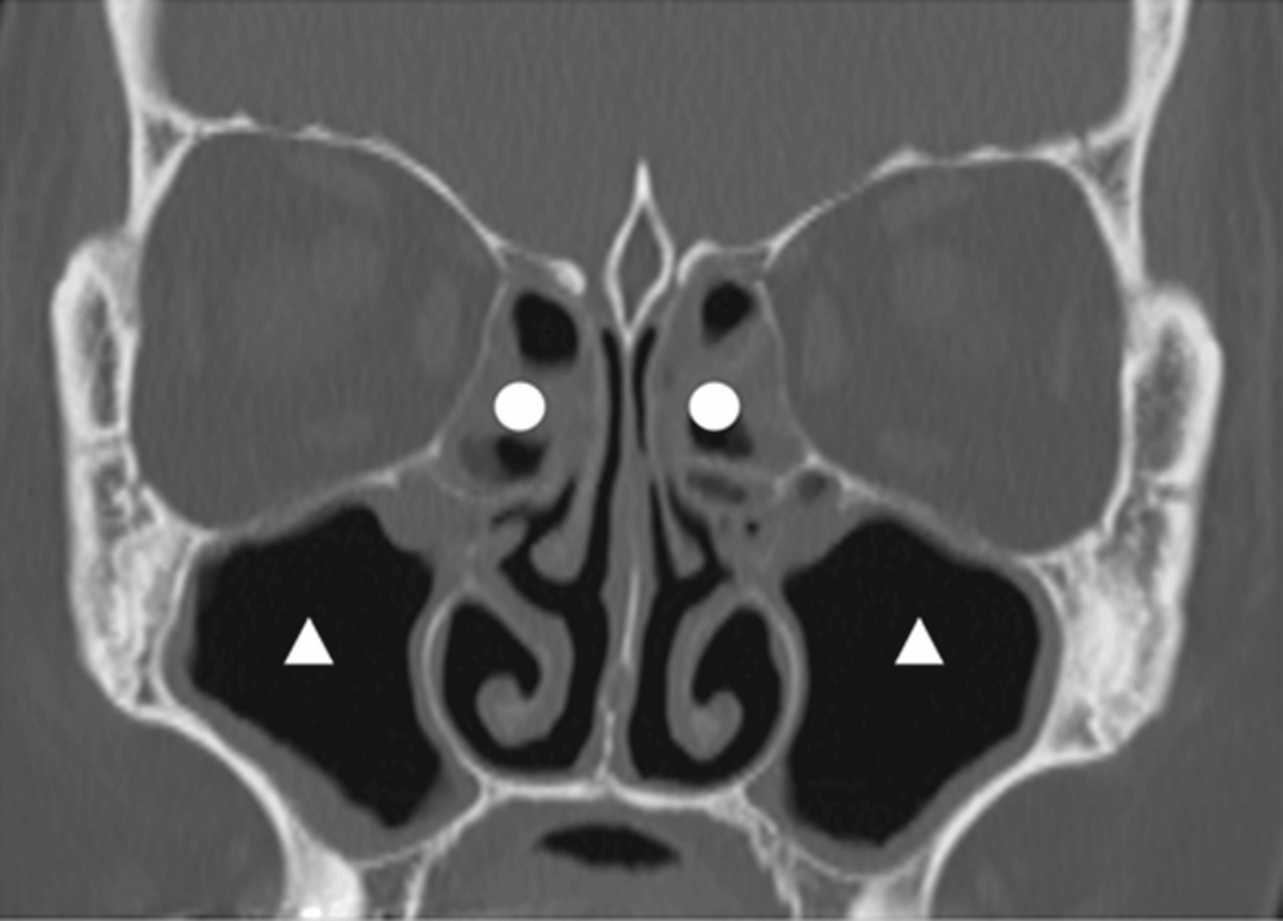
Sinus CT (coronal section) Opacities were more prominent in the ethmoid sinuses (〇) than in the maxillary sinuses (△).

A 58-year-old woman presented with persistent post-nasal drip and coughing. Her medical history included bronchial asthma and depression, and known allergies to Japanese cedar and cypress pollen. Despite receiving treatment for bronchial asthma with oral corticosteroids and inhalers over the past year, the symptoms remained poorly controlled. The presence of post-nasal drip raised suspicion of coexisting eosinophilic chronic rhinosinusitis (ECRS). Endoscopic sinus surgery was initially scheduled for her at another hospital but was canceled on the day of surgery due to an exacerbated asthma attack. She subsequently sought treatment at our facility, leading to a detailed reevaluation of her condition. Nasal endoscopy revealed no obvious polyps in either nostril. Computed tomography of the paranasal sinuses revealed extensive soft tissue opacities in the bilateral paranasal sinuses, which were more prominent in the ethmoid sinuses compared to the maxillary sinuses (Figure 1). Blood tests revealed an eosinophil count of 8% in peripheral white blood cells. Based on these findings, the patient’s JESREC score was 13, suggesting ECRS [5]. However, the absence of nasal polyps made a definitive diagnosis challenging before surgery. Based on these findings, oral corticosteroid was discontinued, and surgery was planned for both diagnostic and therapeutic purposes. Preoperative spirometry results were normal; however, since the test was conducted while the patient was taking oral corticosteroids, it may not have accurately reflected their true pulmonary function. Oral corticosteroid administration was discontinued two weeks prior to surgery.

**Figure 2:**
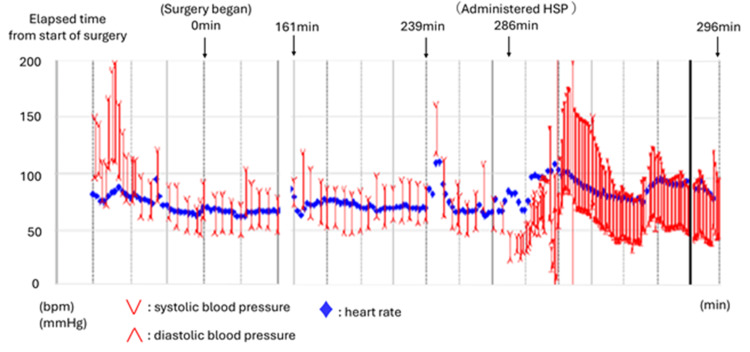
Intraoperative course An increase in airway pressure, suspected to be due to an asthma attack, was observed (161 minutes after surgery initiation). The surgery was completed (239 minutes after surgery initiation). HSP rapidly administered intravenously to prevent asthma attacks (286 min after the initiation) resulted in a sudden increase in airway pressure, leading to complete ventilation failure (289 minutes after surgery initiation). Owing to stabilized circulation and respiratory status, the patient was admitted to the ICU while still intubated (296 min after surgery initiation).

During endoscopic sinus surgery, an increase in airway pressure, suspected to be an asthma attack, was observed and improved following inhaled procaterol administration (161 min after surgery initiation). Sugammadex was administered at the end of surgery (239 min after surgery initiation). However, nasal bleeding was observed immediately before extubation, necessitating a hemostasis procedure. During hemostasis, hydrocortisone sodium phosphate (HSP) was rapidly administered intravenously to prevent asthma attacks (286 min after surgery initiation), which resulted in a sudden increase in airway pressure, leading to complete ventilation failure (289 min after surgery initiation). Systolic blood pressure dropped to 40 mmHg, and SpO2 decreased to 70% (FiO2, 0.47). Procaterol inhalation produced no improvement, necessitating intramuscular and continuous intravenous adrenaline administration to stabilize both the circulatory and respiratory status. She was admitted to the intensive care unit (ICU) while remaining intubated (296 min after surgery initiation) (Figure 2).

Postoperatively, the patient was treated with procaterol inhalation, intravenous betamethasone, and aminophylline. The patient’s respiratory and circulatory status improved rapidly, and she was extubated one day after surgery. She was transferred to the general ward on postoperative day 4 and was discharged on postoperative day 7. A histopathological examination confirmed eosinophilic infiltration of the ethmoid sinus mucosa (eosinophil count: 536/HPF), meeting the diagnostic criteria for ECRS.

The following medications were administered perioperatively: propofol, fentanyl, remifentanil, rocuronium, cefazolin, sugammadex, and HSP. Given the sudden deterioration in the patient’s condition, anaphylactic shock was suspected, thought to be triggered by either sugammadex or HSP. A skin prick test was performed to identify causative agents. The skin prick test revealed the development of wheals and erythema on the skin in response to HSP, with a wheal diameter approximately half that of the positive control (10 mg/mL histamine dihydrochloride), which confirmed a positive result [10]. No reaction was observed with sugammadex.

This case report was conducted in accordance with the principles of the Declaration of Helsinki. Written informed consent was obtained from the patient for the publication of this case report, including all accompanying images. The patient was assured that her identity would remain anonymous and no identifiable personal information would be included. Institutional ethical approval was not required, as this was a single case report without experimental interventions.

## Discussion

N-ERD is a non-allergic hypersensitivity reaction characterized by severe respiratory symptoms, including nasal obstruction, rhinorrhea, and asthma attacks, triggered by NSAIDs that inhibit cyclooxygenase (COX)-1, an enzyme involved in prostaglandin (PG) synthesis. The pathogenesis of N-ERD differs significantly from that of allergic reactions like anaphylaxis [11-14]. N-ERD is most commonly induced by NSAIDs with COX-1 inhibitory activity, whereas selective COX-2 inhibitors, such as celecoxib, are generally safe for use. This suggests that this condition represents non-allergic hypersensitivity specific to COX-1 inhibitors [11,12]. While the underlying mechanism remains unclear, various triggers, including anti-inflammatory drugs and antipyretic analgesics (particularly NSAIDs), food and pharmaceutical additives such as tartrazine (colorant), sodium benzoate (preservative), and parabens (preservatives), and SSC, have been reported to induce N-ERD attacks [9,15].

In this case, anaphylactic shock caused by HSP was considered, as indicated by a positive skin prick test. Although there have been numerous reports regarding anaphylactic shock due to SSC, cases of anaphylactic shock due to SPC are rare. Reports have also described asthma attacks presenting with anaphylaxis-like symptoms following corticosteroid administration [16]. Given that anaphylaxis and asthma attacks can present with similar symptoms, distinguishing between the two can be challenging. Since increased airway pressure is also observed in asthma attacks, the actual condition in this case was possibly due to an asthma attack.

Corticosteroids are sometimes administered during severe asthma exacerbations. Although SPCs are generally considered safe for patients with N-ERD [9], asthma attacks may also be triggered by drug additives [15]. In this case, the HSP formulation used intraoperatively contained hydrocortisone sodium phosphate as the active ingredient, with the following additives: creatinine (stabilizer), sodium bisulfite (preservative), methylparaben (preservative), propylparaben (preservative), sodium citrate dihydrate (pH adjuster), and sodium hydroxide (pH adjuster). Although HSP is classified as SPC, it contains parabens. Therefore, the N-ERD in this case may have been induced by the additives contained in the HSP. Sugammadex does not contain any additives that could potentially trigger N-ERD and was therefore deemed unrelated to the observed acute deterioration. Similarly, betamethasone, administered postoperatively and also classified as an SPC, does not contain any additives that could induce N-ERD and was therefore considered to have no effect.

Additionally, rapid intravenous administration of corticosteroids has been reported to exacerbate asthma attacks, whereas administering corticosteroids over a period of more than 2 hours is considered safer [9]. The rapid administration of corticosteroid (HSP) may have contributed to the induction of N-ERD in this case. Although asthma attacks triggered by SPC are rare, it is recommended that SPC be administered over a period of 1-2 hours, even when it is used to treat N-ERD. Rapid intravenous administration of substances that can trigger N-ERD without considering the patient's condition can result in fatal asthma attacks. Therefore, clinicians should consider this possibility when managing patients with N-ERD.

The patient was receiving oral corticosteroids (prednisolone) prior to visiting our hospital. Since oral corticosteroids have a chemical structure almost identical to that of endogenous corticosteroids, they are not considered N-ERD triggers [9]. Furthermore, preoperative interviews reported no history of NSAID-induced asthma attacks. However, NSAID hypersensitivity is notably acquired later in life [17]. Spector et al. reported that 15 of 171 patients with asthma without a history of aspirin hypersensitivity (8.8%) tested positive for aspirin provocation [18]. This suggests that N-ERD may not be diagnosed through preoperative history.

N-ERD is often associated with severe corticosteroid-dependent asthma [12], and 90-100% of patients with N-ERD develop ECRS [19]. Therefore, N-ERD should be suspected in patients with severe asthma and olfactory dysfunction, even without a history of NSAID-induced asthma attacks. The patient had a history of severe corticosteroid-dependent asthma. There was no olfactory dysfunction, but ECRS was suspected, so N-ERD should have been considered.

Effective bronchial asthma controlled preoperatively may have prevented the acute deterioration observed in this case. However, this is not always feasible. Recognizing the possibility of N-ERD and administering appropriate medications can help prevent life-threatening attacks.

## Conclusions

This report presented a case in which an asthma attack occurred during surgery for ECRS, with N-ERD suspected to have been induced by the additives of SPC. Although intravenous SPC administration is generally considered safe for N-ERD, certain additives in these medications may trigger the condition. While NSAIDs are the most recognized triggers of N-ERD, other triggers should also be considered. N-ERD is often associated with severe corticosteroid-dependent asthma, and most N-ERD patients develop ECRS. Therefore, N-ERD should be suspected in patients with severe asthma and olfactory dysfunction, even without a history of NSAID-induced asthma attacks. It is important to confirm symptoms and medical history during the interview. Furthermore, rapid intravenous administration of potential triggers of N-ERD without recognizing the condition can lead to life-threatening asthma attacks. Slow administration of these drugs is recommended, and clinicians should consider the possibility of N-ERD when managing patients.
